# Alcoholic Liver Disease: Pathogenesis and Current Management

**DOI:** 10.35946/arcr.v38.2.01

**Published:** 2017

**Authors:** Natalia A. Osna, Terrence M. Donohue, Kusum K. Kharbanda

**Affiliations:** Natalia A. Osna, Ph.D., is a Research Biologist in the Research Service, Veterans Affairs Nebraska-Western Iowa Health Care System, and an Associate Professor in the Department of Internal Medicine, University of Nebraska Medical Center, both in Omaha, Nebraska. Terrence M. Donohue, Jr., Ph.D., is a Research Biochemist in the Research Service, Veterans Affairs Nebraska-Western Iowa Health Care System, and a Professor in the Departments of Internal Medicine and Biochemistry and Molecular Biology, University of Nebraska Medical Center, both in Omaha, Nebraska. Kusum K. Kharbanda, Ph.D., is a Research Biologist in the Research Service, Veterans Affairs Nebraska-Western Iowa Health Care System, and a Professor in the Departments of Internal Medicine and Biochemistry and Molecular Biology, University of Nebraska Medical Center, both in Omaha, Nebraska

**Keywords:** Alcohol consumption, heavy drinking, alcohol effects and consequences, abstinence, alcoholic liver disease, liver injury, hepatic lesions, steatosis, hepatitis, fibrosis, cirrhosis, treatment, pharmacological therapy, nutritional therapy, liver transplantation

## Abstract

Excessive alcohol consumption is a global healthcare problem. The liver sustains the greatest degree of tissue injury by heavy drinking because it is the primary site of ethanol metabolism. Chronic and excessive alcohol consumption produces a wide spectrum of hepatic lesions, the most characteristic of which are steatosis, hepatitis, and fibrosis/cirrhosis. Steatosis is the earliest response to heavy drinking and is characterized by the deposition of fat in hepatocytes. Steatosis can progress to steatohepatitis, which is a more severe, inflammatory type of liver injury. This stage of liver disease can lead to the development of fibrosis, during which there is excessive deposition of extracellular matrix proteins. The fibrotic response begins with active pericellular fibrosis, which may progress to cirrhosis, characterized by excessive liver scarring, vascular alterations, and eventual liver failure. Among problem drinkers, about 35 percent develop advanced liver disease because a number of disease modifiers exacerbate, slow, or prevent alcoholic liver disease progression. There are still no FDA-approved pharmacological or nutritional therapies for treating patients with alcoholic liver disease. Cessation of drinking (i.e., abstinence) is an integral part of therapy. Liver transplantation remains the life-saving strategy for patients with end-stage alcoholic liver disease.

Excessive alcohol consumption is a global healthcare problem with enormous social, economic, and clinical consequences, accounting for 3.3 million deaths in 2012 (World Health Organization 2014). Excessive drinking over decades damages nearly every organ in the body. However, the liver sustains the earliest and the greatest degree of tissue injury from excessive drinking because it is the primary site of ethanol metabolism (Lieber 2000). After a brief overview of alcohol metabolism in the liver, this article will summarize the mechanisms through which excessive alcohol consumption contributes to the development of various types of alcohol-induced liver damage. It also will review modifiers of alcoholic liver disease (ALD) and discuss currently used treatment approaches for patients with ALD.

## Hepatic Alcohol Metabolism

**Figure 1:**
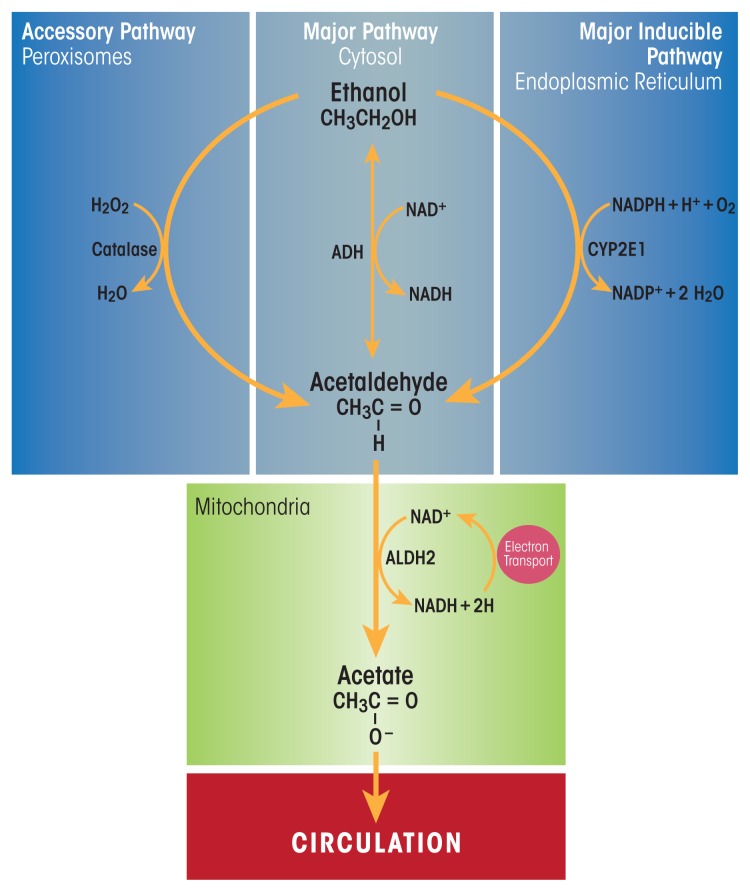
Major and minor ethanol-oxidizing pathways in the liver. Ethanol (i.e., ethyl alcohol) is oxidized principally in hepatocytes of the liver. **(Middle panel)** Alcohol dehydrogenase (ADH), a major enzyme in the cytosol, and aldehyde dehydrogenase 2 (ALDH2), which is located in the mitochondria, catalyze sequential oxidations that convert ethanol to acetate, producing two mole equivalents of reduced nicotinamide adenine dinucleotide (NADH). **(Right panel)** Cytochrome P450 2E1 (CYP2E1) is a major inducible oxidoreductase in the endoplasmic reticulum that oxidizes ethanol, in the presence of molecular oxygen (O_2_), to acetaldehyde and converts reduced NAD phosphate (NADPH) to its oxidized form, generating water. **(Left panel)** Peroxisomal catalase is a minor hepatic pathway of ethanol oxidation that uses hydrogen peroxide (H_2_O_2_) to oxidize ethanol to acetaldehyde and water. SOURCE: Figure adapted from Zakhari and Li 2007.

Beverage alcohol (i.e., ethanol) is chiefly metabolized in the main parenchymal cells of the liver (i.e., hepatocytes) that make up about 70 percent of the liver mass (Jones 1996). These cells express the highest levels of the major ethanol-oxidizing enzymes, alcohol dehydrogenase (ADH), which is located in the cytosol, and cytochrome P450 2E1 (CYP2E1), which resides in the smooth endoplasmic reticulum (ER) (figure 1). Hepatocytes also express very high levels of catalase, an enzyme that inhabits peroxisomes. Catalase normally carries out the detoxification of hydrogen peroxide (H_2_O_2_) to water and oxygen. However, when ethanol is present, catalase has an accessory role in ethanol metabolism by using H_2_O_2_ to oxidize ethanol to acetaldehyde. Ethanol oxidation by catalase is a relatively minor pathway in the liver, but has a larger ethanol-oxidizing function in the brain (Aragon et al. 1992).

ADH is the most catalytically efficient ethanol-metabolizing enzyme. It reaches its half-maximal velocity when circulating ethanol levels are about 5 to 10 milligrams per deciliter, well below levels that cause intoxication.^1^ ADH-catalyzed ethanol oxidation uses nicotinamide adenine dinucleotide (NAD^+^) as a cofactor, generating reduced NAD^+^ (NADH) and acetaldehyde. The latter compound is highly reactive and toxic. It can covalently bind to proteins (Donohue et al. 1983), lipids (Kenney 1982), and nucleic acids (Brooks and Zakhari 2014) to form acetaldehyde adducts, which, in turn, can disrupt the structure and function of these macromolecules (Mauch et al. 1986). One way that hepatocytes minimize acetaldehyde toxicity is by rapidly oxidizing it to acetate using the enzyme aldehyde dehydrogenase 2 (ALDH2) inside mitochondria. The ALDH2 reaction is another oxidation–reduction step that generates NADH and acetate, the latter of which can diffuse into the circulation to be utilized in other metabolic pathways. The enhanced generation of NADH by both ADH- and ALDH2-catalyzed reactions decreases the normal intrahepatocyte NAD^+^/NADH ratio, called the cellular redox potential. This change causes significant metabolic shifts from oxidative metabolism toward reductive synthesis, favoring the formation of fatty acids, which contribute to fatty liver development (Donohue 2007).

CYP2E1 is the other major hepatic enzyme that catalyzes ethanol oxidation to acetaldehyde. Although the catalytic efficiency of CYP2E1 is considerably slower than that of ADH, CYP2E1 has a 10-fold higher capacity for binding ethanol, becoming half-saturated at 46 to 92 milligrams per deciliter. Also important is that CYP2E1 is an inducible enzyme; its hepatocellular content rises during chronic ethanol consumption (Dilger et al. 1997; Lieber and DeCarli 1968). Ethanol interacts directly with the CYP2E1 protein, causing it to assume a conformation that resists degradation by the ubiquitin-proteasome system and resulting in the accumulation of CYP2E1 molecules (Roberts et al. 1995). CYP2E1 induction has several major effects in heavy drinkers: First, because more CYP2E1 oxidizes ethanol, drinkers develop a “metabolic tolerance”—that is, they need to drink more alcohol to reach a level of intoxication that they formerly achieved after drinking less alcohol. Second, accelerated alcohol metabolism by higher levels of CYP2E1 puts liver cells in metabolic peril, because more CYP2E1 not only produces more acetaldehyde, but the induced enzyme also generates greater amounts of various other reactive oxygen species (ROS), including hydroxyethyl radicals (i.e., free-radical forms of ethanol), superoxide anions (O_2_^−^) and hydroxyl radicals (·OH). Continuous generation of these reactive molecules in problem drinkers eventually creates the condition known as oxidant stress or oxidative stress. Under these conditions, the rate of ROS generation exceeds the liver’s capacity to neutralize them with natural antioxidants, such as glutathione and vitamins E, A, and C, or to remove them using antioxidant enzymes, including those listed in table 1 (Fang et al. 2002). Animal studies have revealed that chronic ethanol consumption decreases the activities and/or amounts of several antioxidant enzymes, which worsens the hepatocytes’ oxidant burden (Chen et al. 1995; Dong et al. 2014; Zhao et al. 1996). Oxidant stress further is exacerbated when the generated ROS undergo secondary reactions with proteins and unsaturated lipids. The latter reactions result in the generation of lipid peroxides, which themselves interact with proteins and with acetaldehyde to form bulkier adducts (e.g., malondialdehyde-acetaldehyde [MAA] adducts) that are capable of generating an immune response (Tuma et al. 1996). Finally, because of CYP2E1’s broad substrate specificity, increased levels of the enzyme also accelerate the conversion of excess amounts of substrates other than ethanol, such as the analgesic and antipyretic medication acetaminophen. Following CYP2E1 induction by heavy drinking, acetaminophen is converted to a more toxic, reactive intermediate. This places the chronic drinker at substantial risk for liver disease or acute liver failure, especially after an acetaminophen overdose (Schiodt et al. 2002).

### Alcohol’s Effects on Other Liver Cell Types

Although hepatocytes comprise most of the liver mass, nonparenchymal cells, including Kupffer cells (KCs), sinusoidal endothelial cells, hepatic stellate cells (HSCs), and liver-associated lymphocytes make up the remaining 15 to 30 percent of the liver mass. These nonparenchymal cells interact with hepatocytes and with each other via soluble mediators and by direct cell-to-cell contact. Each liver cell type plays a specific role not only in normal hepatic physiology but also in initiating and perpetuating liver injury.

## Spectrum of ALD

**Figure 2:**
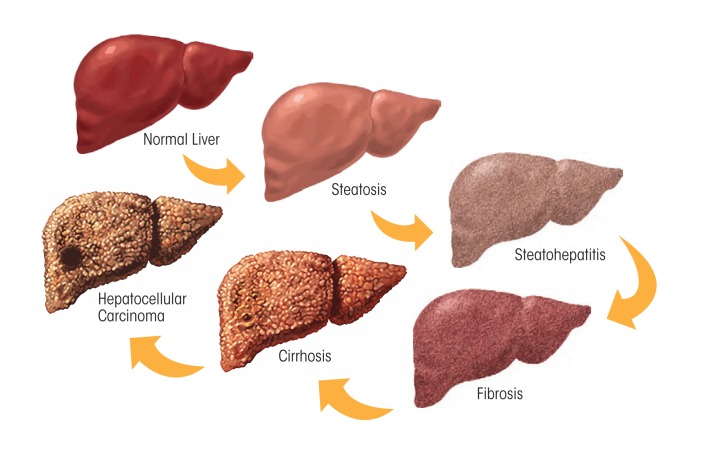
Spectrum of alcoholic liver disease. Heavy ethanol consumption produces a wide spectrum of hepatic lesions. Fatty liver (i.e., steatosis) is the earliest, most common response that develops in more than 90 percent of problem drinkers who consume 4 to 5 standard drinks per day. With continued drinking, alcoholic liver disease can proceed to liver inflammation (i.e., steatohepatitis), fibrosis, cirrhosis, and even liver cancer (i.e., hepatocellular carcinoma).

**Figure 3:**
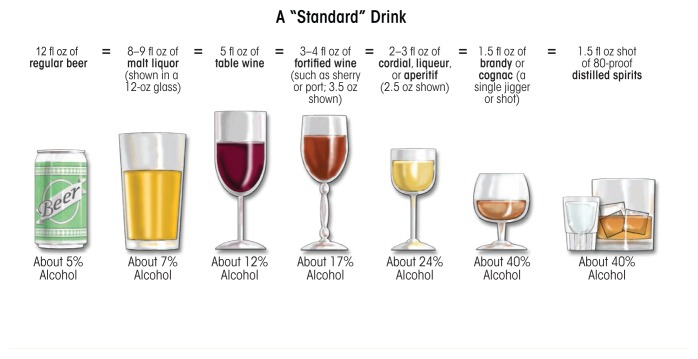
Illustration of “standard drinks” in order of increasing ethanol content among currently available alcoholic beverages. According to the National Institute on Alcohol Abuse and Alcoholism, the amount of beverage containing approximately 14 g of pure ethanol is defined as a standard drink. The percent of pure alcohol, expressed as alcohol by volume (alc/vol), varies by beverage. Thus, 12 ounces (360 mL) of beer at 6 percent alc/vol, 5 ounces (150 mL) of wine at 12 percent alc/vol, or 1.5 ounces (45 mL) of distilled spirits at 40 percent alc/vol each are equivalent to a standard drink. Although the standard-drink amounts are helpful for following health guidelines, they may not reflect customary serving sizes. In addition, although the alcohol concentrations listed are typical, there is considerable variability in actual alcohol content within each type of beverage.

Heavy ethanol consumption produces a wide spectrum of hepatic lesions, the most characteristic being fatty liver (i.e., steatosis), hepatitis, and fibrosis/cirrhosis (see figure 2). Steatosis is the earliest, most common response that develops in more than 90 percent of problem drinkers who consume 4 to 5 standard drinks per day over decades (Ishak et al. 1991; Lieber 2004). (A standard drink is defined as the amount of alcoholic beverage that contains approximately 0.5 fluid ounces, or about 14 grams, of pure alcohol [figure 3]). However, steatosis also develops after binge drinking, defined as the consumption of 4 to 5 drinks in 2 hours or less. Steatosis was formerly considered a benign consequence of alcohol abuse. It is characterized by the deposition of fat, seen microscopically as lipid droplets, initially in the hepatocytes that surround the liver’s central vein (i.e., perivenular hepatocytes), then progressing to mid-lobular hepatocytes, and finally to the hepatocytes that surround the hepatic portal vein (i.e., periportal hepatocytes). If the affected individual ceases drinking, steatosis is a reversible condition with a good prognosis. However, patients with chronic steatosis are more susceptible to fibrotic liver disease (Teli et al. 1995), because the presence of fat likely represents a greater risk for lipid peroxidation and oxidative damage.

Alcoholic hepatitis is a more severe, inflammatory type of liver injury characterized by swollen, dying hepatocytes (i.e., ballooning degeneration), neutrophilic infiltration, and the development of tangled aggregates of insoluble proteins called Mallory-Denk bodies within hepatocytes. Central to hepatitis development is the activation of KCs, the resident liver macrophages.

Fibrosis and its terminal or late stage, cirrhosis, refer to the deposition of abnormal amounts of extracellular matrix proteins, principally by activated HSCs. Patients initially exhibit active pericellular fibrosis, which may progress to cirrhosis, the late stage of hepatic scarring. However, some degree of hepatitis likely is always present in cirrhotic patients, whereas hepatic fat usually is not prominent in these individuals. The World Health Organization’s (2014) *Global Status Report on Alcohol and Health* estimates that 50 percent of all deaths caused by cirrhosis were attributable to alcohol abuse.

The following sections provide a detailed description of the mechanisms involved in the development of these major lesions.

## Mechanisms Involved in Alcoholic Steatosis

**Figure 4:**
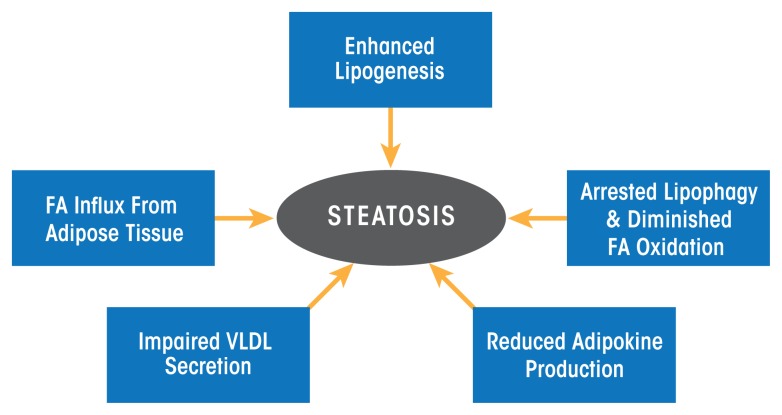
Hepatic and extrahepatic mechanisms that contribute to the development of alcoholic fatty liver (i.e., steatosis). NOTE: FA = fatty acid; VLDL = very low density lipoprotein.

As the preceding section on ethanol metabolism stated, ethanol and acetaldehyde oxidations generate higher levels of NADH, which alters the cellular redox potential and enhances lipid synthesis (i.e., lipogenesis). However, ethanol-induced redox change alone does not fully explain why the liver rapidly accumulates fat. More recent studies now strongly support the notion that ethanol-induced steatosis is multifactorial as discussed below (see figure 4).

### Alcohol Accelerates Hepatic Lipogenesis

**Figure 5:**
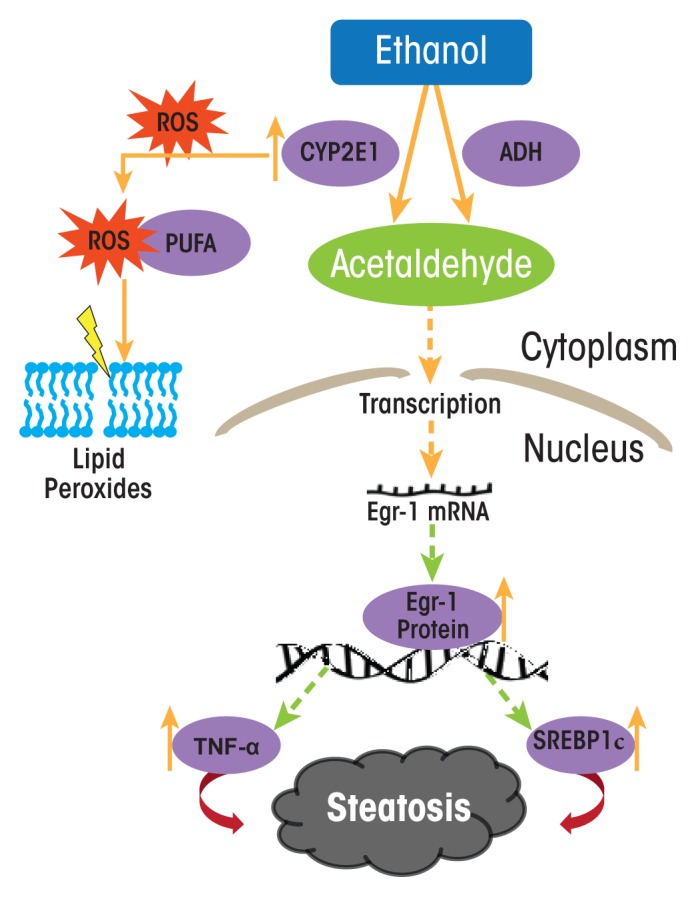
Proposed mechanism by which ethanol oxidation regulates early growth response-1 (Egr-1) and sterol regulatory element binding protein-1c (SREBP-1c) to enhance lipogenesis. Alcohol dehydrogenase (ADH) and cytochrome P450 2E1 (CYP2E1) each catalyze ethanol oxidation, producing acetaldehyde. This aldehyde enhances Egr-1 gene transcription by activating the Egr-1 promoter, thereby increasing the levels of Egr-1 mRNA and, subsequently, nuclear Egr-1 protein. It is believed that nuclear Egr-1 protein regulates transcription of SREBP-1c and tumor necrosis factor (TNF) genes to initiate ethanol-induced lipogenesis and fatty liver (i.e., steatosis). NOTE: PUFA = polyunsaturated fatty acid; ROS = reactive oxygen species. SOURCE: Figure adapted from Thomes et al. 2013.

Enhanced lipid synthesis results from a higher expression of lipogenic enzymes and cytokines (see table 2) that are encoded by genes regulated by two transcription factors, sterol regulatory element binding protein-1c (SREBP-1c) and early growth response-1 (Egr-1). SREBP-1c belongs to a family of transcription factors that control hepatic cholesterol metabolism. However, in heavy drinkers, ethanol oxidation short-circuits hepatic lipid metabolism, converting the liver from a lipid-burning to a lipid-storing organ. Thus, hepatic SREBP-1c is relatively inactive in hepatocytes of abstinent people, residing mostly in the ER. However, in a person who binges or habitually drinks, hepatic ethanol oxidation triggers the translocation of SREBP-1c from the ER to the Golgi apparatus, where it undergoes proteolytic maturation to its active form, generating a transcriptionally active SREBP protein fragment that enters the nucleus and enhances lipogenic gene expression (see table 2). Egr-1 controls the expression of genes that respond to cellular stress. It binds to gene promoter regions that are relevant to alcohol-induced liver injury and steatosis. The most notable of these is tumor necrosis factor alpha (TNFα), a lipogenic cytokine. Additionally, because Egr-1 is activated very early after ethanol administration (Donohue et al. 2012), it also regulates the expression of the SREBP-1c gene (Thomes et al. 2013). Figure 5 shows the postulated scheme of transcriptional control that contributes to enhanced lipogenesis in the liver.

In addition to enhanced hepatic lipogenesis, fat (i.e., adipose) tissue contributes to the development of steatosis. Adipose tissue normally is an important energy depot, storing excess calories derived from food consumption as fat. When necessary, high-energy fat then can be used to fulfill energy requirements during times of low nutrition (e.g., fasting) or high calorie utilization (e.g., exercise). Research with rodents subjected to chronic alcohol feeding has shown that ethanol consumption reduces adipose tissue mass by enhancing fat breakdown (i.e., lipolysis) in adipose tissue (Kang et al. 2007; Wang et al. 2016; Wei et al. 2013). The free fatty acids released from adipose tissue are taken up by the liver and esterified into triglycerides, thereby exacerbating fat accumulation in the liver (Wei et al. 2013). Clinical studies also have demonstrated that people with alcohol use disorder who have fatty liver have significantly lower body weight, body mass index, and body-fat mass content than control subjects (Addolorato et al. 1997, 1998).

### Alcohol Decelerates Hepatic Lipid Breakdown

Because most lipids in hepatocytes are stored in lipid droplets, these organelles must first be degraded to extract the lipids for their subsequent oxidation. Breakdown of lipid droplets is accomplished by lipophagy, a specialized form of the intracellular process that degrades cytoplasmic components (i.e., autophagy). During lipophagy, lipid droplets are engulfed within double- membrane–bound vacuoles called autophagosomes. These vacuoles transport the lipid-droplet cargo to lysosomes, where they are degraded by lipid-digesting enzymes (i.e., lipases), releasing free fatty acids that then undergo β-oxidation inside mitochondria. The rates of autophagy reportedly are retarded by chronic ethanol consumption, at least in part because ethanol is thought to cause faulty lysosome biogenesis. This results in fewer, more defective lysosomes (Kharbanda et al. 1995, 1996), thereby slowing the breakdown of lipid droplets in the steatotic liver.

It also is quite clear that once fatty acids are released from lipid droplets, heavy alcohol consumption reduces their rates of β-oxidation. There are several reasons for the slowdown: First, the enhanced generation of NADH by ethanol oxidation inhibits mitochondrial β-oxidation. Second, metabolically generated acetaldehyde inactivates the peroxisome proliferator activated receptor alpha (PPAR-α), a transcription factor that acts in concert with the retinoid X receptor (RXR) and governs expression of genes that regulate fatty-acid transport and oxidation. Acetaldehyde likely inactivates PPAR-α by covalently binding to the transcription factor (Galli et al. 2001), thereby blocking its ability to recognize and/or bind PPAR-α promoter sequences. Third, both acute and chronic ethanol oxidation cause mitochondrial depolarization, impairing their abilities to generate energy (i.e., adenosine triphosphate [ATP] molecules), and causing their outer membranes to leak, resulting in inefficient fatty-acid import and lower rates of β-oxidation (Zhong et al. 2014). Fourth, ethanol consumption reduces the production of the hormone adiponectin, which is secreted by fat cells (i.e., adipocytes). One study demonstrated that the restoration of adiponectin to alcohol-fed animals re-establishes fatty-acid oxidation to normal (Xu et al. 2003). In addition, adiponectin appears to reduce the production of the cytokine TNFα, and there is evidence that TNFα also may regulate adiponectin production (You and Crabb 2004).

### Alcohol Causes Defective Hepatic Lipid Export

It is well known that the liver exports triglycerides and cholesterol only as constituents of very low density lipoprotein (VLDL) particles; any impairment in either the synthesis or export of VLDL particles therefore contributes to fat accumulation within hepatocytes. VLDL assembly is regulated by the availability of triglycerides (which make up more than 50 percent of the VLDL lipids) stored in cytoplasmic lipid droplets. Up to 70 percent of the triglycerides in VLDLs are derived from the pool of triglycerides stored in lipid droplets that first undergo lipolysis and then are re-esterified to constitute VLDL triglycerides. Although earlier reports implicated altered VLDL secretion in the development of alcoholic steatosis (Venkatesan et al. 1988), exactly how alcohol impairs lipolysis of triglyceride stores in lipid droplets for assembly of VLDL and its subsequent secretion is unknown. However, studies have shown that alcohol-impaired VLDL secretion is caused by a decreased synthesis of an essential constituent of VLDL (Kharbanda et al. 2007, 2009) as well as by reduced activity of an essential protein for its assembly (Shearn et al. 2016; Sugimoto et al. 2002).

## Mechanisms Involved in Alcoholic Hepatitis

Alcoholic hepatitis occurs in about 30 to 40 percent of individuals reporting chronic alcohol abuse. It represents the most serious form of ALD and is associated with high short-term mortality. Ballooning degeneration of hepatocytes containing Mallory-Denk bodies, infiltrating neutrophils, and fibrosis are characteristic pathologic findings indicative of hepatitis (Lefkowitch 2005). Central to the progression of alcoholic hepatitis are resident and infiltrating immune cells called macrophages, which have important roles in inducing liver inflammation. KCs, the resident macrophages in the liver, represent up to 15 percent of liver cells and 50 percent of all macrophages in the body. They reside in the liver sinusoids and provide the first line of defense, serving as potent innate immune cells. In contrast, infiltrating macrophages are recruited as immature cells from the bone marrow, and their differentiation into macrophages in the liver only occurs during inflammation.

The ability of macrophages to regulate inflammation depends on their polarization—that is, their ability to develop into one of two different functional states, namely M1 (i.e., proinflammatory) or M2 (i.e., anti-inflammatory) macrophages. The polarization to either phenotype depends on the microenvironment, including circulating growth factors, cytokines, and pathogen-associated molecular pattern (PAMP) as well as damage-associated molecular pattern (DAMP) molecules. Because the liver is exposed to countless antigens, pathogens, and toxic substances that come from the intestine via the portal circulation, it must be protected from developing an immune response to such exposure. As a result, KCs usually have tolerogenic properties, meaning that they do not respond to all antigens with an immune response. However, excessive alcohol exposure can switch KCs to a proinflammatory M1 phenotype. Usually, ALD progression from liver steatosis to inflammation requires a second insult in addition to the alcohol exposure, such as another toxic insult, nutritional factor, or viral infection (Tsukamoto et al. 2009). More importantly, KCs can regulate the development of inflammation, depending on their ability to either induce or suppress proinflammatory changes. These effects are related to the stage and severity of the alcoholic hepatitis; in severe cases, KCs differentiate to the proinflammatory M1 phenotype, whereas in mild forms, KCs switch to the anti-inflammatory M2 phenotype. As inducers of inflammation, KCs release multiple proinflammatory cytokines, including TNFα, interleukins, and chemokines that attract inflammatory cells from circulation. KCs also are an abundant source of ROS that exacerbate oxidative stress in the liver.

**Figure 6:**
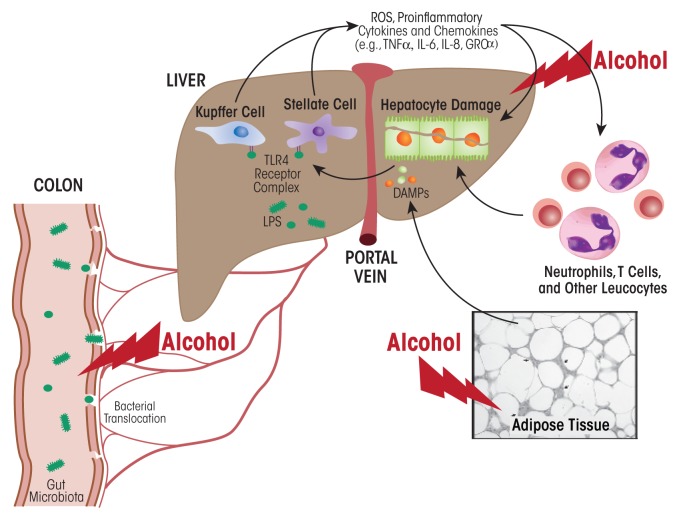
The gut–liver axis. A major factor in the initiation of the inflammatory response by resident macrophages of the liver (i.e., Kupffer cells) is endotoxin or lipopolysaccharide (LPS), a cell-wall component of Gram-negative bacteria that translocates from the gut lumen into the portal circulation to reach the liver. Enhanced circulating endotoxin levels in alcoholic hepatitis are caused by alcohol-induced qualitative and quantitative changes in the bacteria that inhabit the gut (i.e., gut microbiota) and increased gut leakiness. In the liver, LPS activates Kupffer cells and hepatic stellate cells by interacting with toll-like receptor 4 (TLR4). These cells produce reactive oxygen species (ROS) as well as proinflammatory cytokines and chemokines that together with alcohol contribute to hepatocyte damage. Other factors contributing to hepatocyte damage include alcohol-induced activation of various immune cells (i.e., neutrophils, T cells, and other leukocytes) as well as alcohol’s effects on the fat (i.e., adipose) tissue, which results in the production of damage-associated molecular pattern (DAMP) molecules.

What factors trigger KC activity in patients with alcohol use disorder? One major factor is endotoxin, also called lipopolysaccharide (LPS), a cell-wall component of Gram-negative bacteria that translocates from the gut lumen into the portal circulation to reach the liver (figure 6). Accumulating data demonstrate that excess ethanol intake induces endotoxemia through two main mechanisms—by stimulating bacterial overgrowth and by increasing intestinal permeability (Bode and Bode 2003). Animal studies have revealed that increased circulating endotoxin levels correlate with the severity of liver disease (Mathurin et al. 2000). LPS is sensed by two types of receptors—CD14 and toll-like receptor 4 (TLR4)—on the KC surface (Suraweera et al. 2015). These receptors activate KCs to produce proinflammatory cytokines and promote free-radical formation via induction of the reduced nicotinamide adenine dinucleotide phosphate (NADPH) oxidase and CYP2E1. The resulting reactive oxygen and nitrogen species promote the release of proinflammatory cytokines, which in turn increase inflammasome activation in KCs and the release of chemokines that attract circulating immune cells to the liver. Inflammasomes are innate immune-system sensors that regulate the activation of caspase-1 and induce inflammation in response to microbial/ viral pathogens, molecules derived from host proteins, and toxic insults (e.g., alcohol exposure).

Other factors can exacerbate liver inflammation. Prominent among these are MAA adducts that are produced in alcohol-exposed hepatocytes. These adducts are taken up by scavenger receptors on KCs (Ambade and Mandrekar 2012), further promoting the proinflammatory response. Also, because macrophages metabolize ethanol via CYP2E1, the induction of oxidative stress by alcohol exposure activates macrophage-dependent release of proinflammatory cytokines, including TNFα. Although hepatocytes normally are resistant to TNFα, alcohol exposure sensitizes them to the cytokine, causing their death via apoptosis. The resulting release of small vesicles (i.e., exosomes) from dying hepatocytes provides activation signals to KCs (Nagy et al. 2016). Apoptotic hepatocytes are engulfed by KCs, thereby switching their phenotype to M1, which exacerbates inflammation. Inflammation-associated release of chemokines, in turn, attracts circulating macrophages, T-cells, and neutrophils (an additional source of oxidative stress) to the liver. These immune cells, by releasing proinflammatory cytokines and chemokines with direct cytotoxic effects, further promote hepatocyte cell death and the persistence of alcoholic hepatitis.

**Figure 7:**
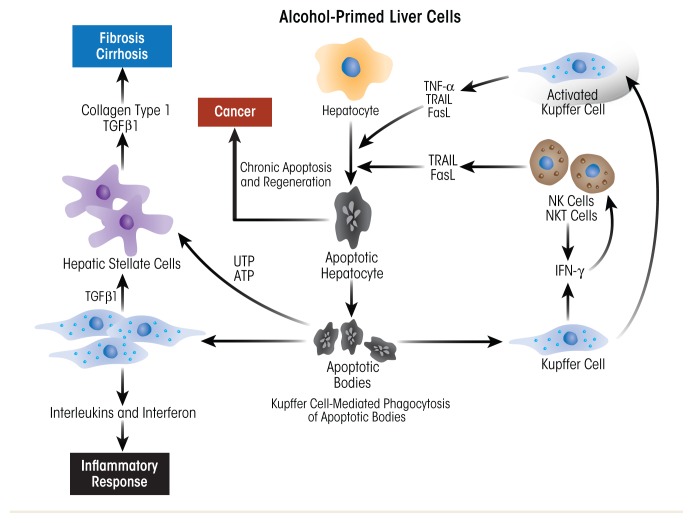
Schematic depiction of the role of Kupffer cells (KCs) and hepatic stellate cells (HSCs) in promoting alcohol-induced inflammatory changes and progression to fibrosis and cirrhosis. Injury begins with alcohol-induced hepatocyte damage and death (apoptosis), which generates apoptotic bodies that stimulate KCs to secrete inflammatory factors, such as tumor necrosis factor alpha (TNFα), interferon gamma (IFN-γ), tumor necrosis factor-related apoptosis-inducing ligand (TRAIL), and Fas ligand (FasL). These factors attract immune cells (e.g., natural killer [NK] cells and natural killer T cells [NKT cells]) to the liver to exacerbate the inflammatory process. Activated HSCs secrete abundant extracellular matrix proteins (e.g., collagen type 1), forming scar tissue (fibrosis) that can progress to cirrhosis. In this condition, the scar tissue forms bands throughout the liver, destroying the liver’s internal structure and impairing the liver’s ability to regenerate itself and to function.

Recently, it was reported that HSCs also play a dual (i.e., stage-dependent) role in the regulation of liver inflammation (Fujita et al. 2016). An important function of HSCs is to transmit signals from sinusoid cells to the liver parenchyma. The proinflammatory cytokines and chemokines produced by activated KCs stimulate the production of proinflammatory cytokines by HSCs. In addition, LPS also can directly activate HSCs through TLR4 to promote the secretion of proinflammatory cytokines. The functions of HSCs are regulated by KCs. The dual role of KCs in the regulation of inflammation is not only related to production of proinflammatory substances. At the stage of the resolution of inflammation, KCs produce anti-inflammatory substances, such as prostaglandin D2, which is sensed by HSC receptors. Prostaglandin D2 programs HSCs to switch their production to anti-inflammatory factors, including transforming growth factor-β1 (TGF-β1), which promotes fibrogenesis. The role of KCs and HSCs in promoting alcohol-induced inflammatory changes and progression to fibrosis/cirrhosis is schematically presented in figure 7.

## Mechanisms Involved in Fibrosis/Cirrhosis

**Figure 8:**
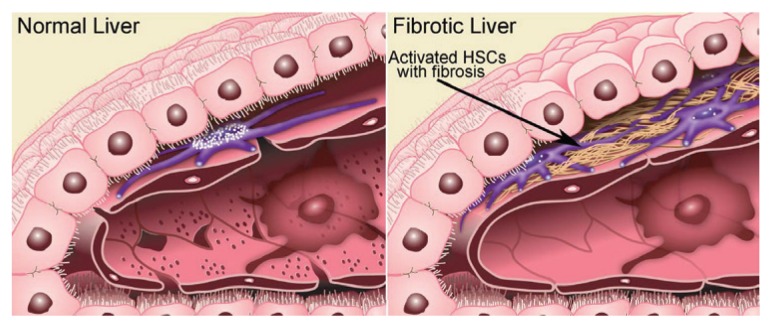
Hepatic stellate cells (HSCs) are key players in the development of fibrosis. HSCs normally reside in the space of Disse as quiescent, lipid (retinyl-ester)-storing cells. Chronic ethanol consumption initiates a complex activation process that transforms these quiescent HSCs into an activated state. Activated HSCs secrete copious amounts of the scar-forming extracellular matrix proteins. This, in turn, contributes to structural changes in the liver, such as the loss of hepatocyte microvilli and sinusoidal endothelial fenestrae, ultimately causing the deterioration of hepatic function. SOURCE: Figure adapted from Friedman 2000.

**Figure 9:**
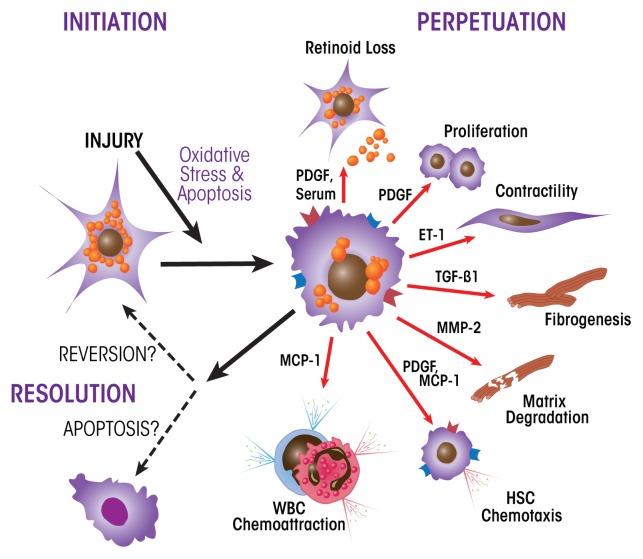
Pathways of hepatic stellate cell (HSC) activation. Following hepatic injury, HSCs undergo a complex activation process involving numerous signaling molecules that is characterized by loss of retinoids, increased proliferation, contractility, and chemotaxis. These activated cells are the principal cell source of increased and irregular deposition of extracellular matrix components, which characterize fibrosis. Activated HSCs also contribute to the inflammatory response by coordinating the recruitment and stimulation of white blood cells (WBCs) by releasing chemokines and proinflammatory cytokines, as well as expressing adhesion molecules. NOTE: ET-1 = endothelin-1; MCP-1 = monocyte chemoattractant protein-1; MMP-2 = matrix metalloproteinase-2; PDGF = platelet-derived growth factor; TGF-β1 = transforming growth factor-beta1. SOURCE: Figure adapted from Friedman 2000.

HSCs are the key players in the development of fibrosis. These cells normally reside in the space of Disse as quiescent, lipid (retinyl-ester)-storing cells (figure 8). Following hepatic injury, HSCs undergo a complex activation process (figure 9) and become the principal source for the increased and irregular deposition of extracellular-matrix components that characterize fibrosis. Activated HSCs also contribute to the inflammatory response, coordinating the recruitment and stimulation of leukocytes by releasing chemokines and proinflammatory cytokines as well as expressing adhesion molecules. The leukocytes, in turn, not only attack and destroy hepatocytes, but also activate quiescent and activated HSCs, thereby exacerbating the fibrogenic response (Friedman 2008).

Hepatic fibrosis is a transient and reversible wound-healing response, which may be restored to normal in some patients if alcohol intake ceases. However, if drinking continues, chronic inflammation and sustained fibrogenesis progress, resulting in the substitution of liver parenchyma by scar tissue that severely compromises the liver’s vascular architecture. The main pathological feature of cirrhosis is the formation of regenerative nodules of hepatic parenchyma surrounded by fibrous septa. Cirrhosis development progresses from a compensated phase, in which part of the liver remains undamaged and functionally compensates for the damaged regions, to a decompensated phase, in which scar tissue fully envelops the organ. The latter is characterized by development of portal hypertension and/or liver failure.

## Modifiers of ALD Risk

Among problem drinkers, only about 35 percent develop advanced liver disease. This is because modifiers, as listed below, exist that exacerbate, slow, or prevent ALD disease progression.

- *Pattern of Consumption and Beverage Type.* The most important factors determining the progression of liver disease are the beverage type consumed and the amount and pattern of drinking (e.g., outside mealtime or binges). Intake of 40 to 80 grams ethanol/day by males and of 20 to 40 grams/day by females for 10 to 12 years is a general predictor of more severe cases of ALD, including alcoholic steatohepatitis, fibrosis, and cirrhosis (Becker et al. 1996).
- *Gender.* Epidemiologic data show that women are more susceptible to alcohol-related liver damage than men. This appears to be related to higher blood alcohol concentrations in women than in men who ingest the same amount of alcohol, resulting from a lower proportion of body water in females compared with males of equal weight (Mumenthaler et al. 1999). There also are reports that women possess a lower capacity than men to oxidize ethanol in the gut, a process called first-pass metabolism (Frezza et al. 1990). This deficit in women allows greater quantities of ethanol into the portal circulation, thereby exposing their livers to higher ethanol concentrations. Further, gender-based differences in the sensitivity of KCs to endotoxins and hepatic inflammatory responses have been related to higher susceptibility to ALD progression in females than in males (Frezza et al. 1990).
- *Age.* It is not completely clear how age modifies ALD progression. It is, however, a predictor for ALD (Masson et al. 2014), because older adults (i.e., ages 65 and up) are more vulnerable to and show greater degrees of ethanol-induced impairments than younger people (Meier and Seitz 2008).
- *Race/Ethnicity.* Ethnicity is a major factor affecting the age at and severity of presentation of different subtypes of ALD (Levy et al. 2015). The reason(s) for these differences are not clear.
- *Genetics.* Both genetic and epigenetic influences govern the initiation and progression of ALD. Genome-wide association studies have identified specific genetic markers (i.e., single-nucleotide polymorphisms) in genes encoding alcohol-metabolizing enzymes, cytokines, and antioxidant enzymes that are related to the progression of ALD (Stickel and Hampe 2012). Most recently, an allele of patatin-like phospholipase domain-containing protein 3 (PNPLA3 I148M), a triglyceride-degrading enzyme, was identified as an independent risk factor for alcoholic cirrhosis (Anstee et al. 2016; Burza et al. 2014).
- *Nutritional Factors.* Dietary fat is a macronutrient and dietary modifier for ALD. In rodents, dietary saturated fat seems to protect against alcohol-induced liver damage, whereas dietary unsaturated fat that is enriched in linoleic acid reportedly promotes such damage (Kirpich et al. 2016).
- *Drugs.* Alcohol and other drugs (including prescription medications, over-the-counter agents, and illicit drugs) interact to enhance hepatotoxicity. For example, as described earlier, acetaminophen hepatotoxicity can be exacerbated by alcohol abuse.
- *Obesity.* Population-based studies have indicated a significant correlation between the risk of liver damage and alcohol consumption in people with a high body mass index (Ruhl and Everhart 2005).
- *Smoking.* Cigarette smoking can adversely affect certain hepatic functions and is associated with higher risk of alcoholic cirrhosis in humans (Klatsky and Armstrong 1992).
- *Viral Infections.* The course of hepatitis C (HCV) and hepatitis B (HBV) viral infections is worsened in alcohol-abusing patients, causing rapid progression to fibrosis, cirrhosis, and even hepatocellular carcinoma (Szabo et al. 2006). Several common mechanisms of viral infection and alcohol-induced damage have been suggested (Zakhari 2013); however, the exact mechanisms for this rapid disease progression are not completely understood. Because viral infections such as HCV or HBV affect more than 170 million people worldwide (Gitto et al. 2014), the following section will describe this topic in greater detail.

### HCV and Alcohol

HCV and alcohol are the two most widespread causes of liver disease worldwide. Almost all patients with a history of both HCV infection and alcohol abuse develop chronic liver injury. Some studies report that 16.9 percent of HCV-infection cases progress to liver cirrhosis, which is twice the prevalence of cirrhosis from alcoholic liver disease. In HCV-positive alcohol abusers, cirrhosis prevalence is even higher at 27.2 percent (Khan and Yatsuhashi 2000). A daily intake of 80 grams of alcohol increases liver-cancer risk 5-fold over that of nondrinkers, whereas heavy alcohol use by HCV-infected individuals increases cancer risk by 100-fold over uninfected heavy drinkers.

There are multiple mechanisms by which alcohol potentiates HCV-infection pathogenesis. For example, HCV proteins induce oxidative stress by binding to the outer membranes of mitochondria, stimulating electron transport and increasing the generation of cellular ROS (e.g., superoxide) (Otani et al. 2005). Coupled with the ethanol-induced depletion of the antioxidant glutathione and ROS-induced suppression of proteasome activity, this compromises cell viability (Osna et al. 2008), causing hepatocyte apoptosis (Ganesan et al. 2015; Siu et al. 2009). Ethanol-induced oxidative stress also causes mutations in the HCV genome that increase resistance to interferon (IFN) treatment, the former standard of care for HCV (Seronello et al. 2011). Only 9 percent of HCV-infected people with alcohol use disorder respond to IFNα therapy. There currently is little information on whether heavy drinking affects the outcomes of HCV treatment with the new generation of antiviral agents (Keating 2015).

Ethanol metabolites appear to stimulate HCV replication. CYP2E1-positive hepatoma cells exposed to ethanol show an increase in HCV RNA (McCartney et al. 2008). However, this rise is only temporarily sustained (Seronello et al. 2007), because these heavily infected cells eventually die by apoptosis (Ganesan et al. 2015). The resulting cell fragments (i.e., apoptotic bodies) contain infectious HCV particles that spread the virus to uninfected cells, causing the production of proinflammatory cytokines by phagocytosing KCs (Ganesan et al. 2016). In addition to apoptotic bodies, another type of cell-derived vesicles (i.e., exosomes) that leak from dead cells enhances intracellular HCV replication in neighboring cells through an exosomal micro-RNA (miRNA 122). Because ethanol exposure also increases hepatic miRNA 122 levels (Bala et al. 2012), HCV replication in problem drinkers likely is augmented (Ganesan et al. 2016).

Innate immunity is the first line of antiviral protection in the liver. HCV commandeers this line of defense, and ethanol metabolism potentiates its takeover. For example, activation of antiviral IFNβ production in liver cells occurs via the interferon regulatory factor 3 pathway, which requires participation of a protein called mitochondrial antiviral signaling protein (MAVS). HCV evades this innate-immunity protection by cleaving MAVS (Gale and Foy 2005), and ethanol metabolism further enhances this cleavage. There are other published examples of how ethanol consumption interferes with the immune response to HCV infection (Ganesan et al. 2015; Siu et al. 2009). Thus, HCV and ethanol synergize in thwarting protective mechanisms that include both innate and adaptive immunity by increasing oxidative stress in liver cells, thereby accelerating the onset of cell death and facilitating the spread of the virus.

## Current Management of ALD

There are no FDA-approved therapies for treating patients with ALD. The following therapies currently are used for optimal ALD management.

### Abstinence

Drinking cessation is considered the most effective therapy in patients with ALD. Abstinence from alcohol not only resolves alcoholic steatosis but also improves survival in cirrhotic patients (Sofair et al. 2010). The effectiveness of abstinence is enhanced when it is combined with lifestyle modifications (e.g., behavioral interventions and dietary alterations) that are supervised by a nurse, primary care physician, or gastroenterologist/hepatologist (Addolorato et al. 2016; Pavlov et al. 2016).

### Natural and Artificial Steroids

Corticosteroid treatment, including the use of prednisolone, has been the most extensively used form of therapy, especially for moderate to severe alcoholic hepatitis, based on their ability to suppress the immune response and proinflammatory cytokine response (Mathurin et al. 1996, 2013; Ramond et al. 1992). However, outcomes with steroids have been variable (Thursz et al. 2015). Current guidelines suggest discontinuation of therapy if there is no indication of a decrease in bilirubin levels by day 7 of treatment (European Association for the Study of the Liver 2012).

### Nutritional Supplements

Nearly all patients with severe alcoholic hepatitis and cirrhosis are malnourished and their degree of malnutrition correlates with disease severity and complications, such as variceal bleeding, ascites, infections, encephalopathy, and hepatorenal syndrome (Halsted 2004; Mendenhall et al. 1995; Stickel et al. 2003). Deficiencies in micronutrients (e.g., folate, vitamin B6, vitamin A, and thiamine) and minerals (e.g., selenium, zinc, copper, and magnesium) often occur in ALD and, in some instances, are thought to be involved in its pathogenesis (Halsted 2004). According to the current guidelines of the American Association for the Study of Liver Diseases, all patients with alcoholic hepatitis or advanced ALD should be assessed for nutritional deficiencies and treated aggressively with enteral nutritional therapy. A protein intake of 1.5 grams per kilogram bodyweight and 35 to 49 kcal per kilogram bodyweight per day is recommended for ALD patients (Frazier et al. 2011). Micronutrient supplementation should be considered if deficiencies are detected. Supplementation with one such micronutrient, zinc, has been shown to be therapeutic in animal models of alcoholic liver injury. Mechanistic studies have revealed that its protection is mediated by blocking or attenuating most mechanisms of liver injury, including increased gut permeability, oxidative stress, increased TNF production, and hepatocyte apoptosis (Mohammad et al. 2012). The few clinical studies conducted to date suggest that zinc supplementation could be an effective therapeutic approach for humans because liver function of ALD and HCV patients improved with 50 mg of elemental zinc (Mohammad et al. 2012).

### Liver Transplantation

This procedure remains the standard of care for patients with end-stage liver disease. Some patients with ALD are not listed for the replacement of their own liver by a donor organ (i.e., orthotopic liver transplantation) for reasons such as continued alcohol consumption, improvement in liver function after abstinence, and a higher incidence of cancers of the upper airways and upper digestive tract. As a result, transplantation candidates with ALD often are screened for common malignancies and must undergo a formal medical and psychiatric evaluation. They also must abstain from alcohol for 6 months before being considered for liver transplantation. Data show that fewer than 20 percent of patients with histories of alcohol use as the primary cause of end-stage liver disease receive liver transplants (Lucey 2014). However, patient and organ survival is excellent in this patient population, with considerable improvement in their quality of life (Singal et al. 2012, 2013). Following transplantation, ALD patients return to consuming alcohol at rates similar to those transplanted for other reasons, although ALD patients may consume greater amounts (Bergheim et al. 2005). Because all transplant recipients exhibit increased levels of alcohol use over time, post-transplant interventions are deemed extremely valuable in supporting patients to maintain abstinence (Donnadieu-Rigole et al. 2017).

### Unconventional and Herbal Remedies

Patients often turn to natural and herbal therapies based on their potential for hepatoprotection. A U.S. survey revealed that 41 percent of patients with liver disease used some form of complementary and alternative medicine. An extract of milk-thistle seeds (silymarin) and garlic were reported as the most commonly used herbs for liver disease, followed by ginseng, green tea, gingko, echinacea, and St. John’s wort (Strader et al. 2002). As indicated in a recent review (Kim et al. 2016), these and other natural medicines, including betaine, curcumin, fenugreek seed polyphenol, LIV-52, vitamin E, and vitamin C, have shown efficacy in experimental models of alcoholic liver injury but must pass the rigors of large randomized, controlled clinical trials.

## Figures and Tables

**Table 1:** Hepatic Enzymatic Defenses Against Free-Radical Attack

Enzyme	Abbreviation	Cellular Location	Function	Effect of Chronic Ethanol Administration	References
**Copper–Zinc-Superoxide Dismutase**	Cu/Zn-SOD	Cytosol	Converts superoxide to H_2_O_2_	Decreases activity and content	Chen et al. 1995; Zhao et al. 1996
**Manganese-Superoxide Dismutase**	Mn-SOD	Mitochondria	Converts superoxide to H_2_O_2_	Decreases activity and content	Chen et al. 1995; Zhao et al. 1996
**Catalase**	Catalase	Peroxisomes	Converts H_2_O_2_ to H_2_O	Increases activity	Chen et al. 1995
**Glutathione Peroxidase**	GSH peroxidase	Cytosol/ mitochondria	Scavenges peroxides and free radicals	Unaffected	Chen et al. 1995
**Glutathione Reductase**	GSSG reductase	Cytosol	Regenerates reduced GSH from GSSG	Decreases activity	Dong et al. 2014
**Glutathione-S-Transferase**	GST	Nuclei, cytosol, mitochondria	Transfers sulfur to acceptor molecules	Increases activity	Chen et al. 1995

**Table 2:** Lipogenic Enzymes Regulated by SREBP-1c

Enzyme	Abbreviation	Function
**Fatty Acid Synthase**	FAS	Synthesizes fatty acids from acetyl coenzyme A (CoA) and palmitate
**Acyl CoA Carboxylase**	ACC	Synthesizes malonyl CoA from acetyl CoA
**ATP Citrate Lyase**	ACL	Converts citrate and CoA to acetyl CoA
**Stearoyl CoA Desaturase**	SCD	Synthesizes unsaturated fatty acids (oleate) from saturated fatty acids (stearate)
**Malic Enzyme**	ME	Generates reducing equivalents (NADPH) for triglyceride synthesis

## Footnotes

1 People are legally inebriated when their blood alcohol levels reach 80 milligrams per deciliter.

## Glossary

Ascites: Accumulation of fluid in the abdominal cavity
Autophagy: The breakdown of organelles (e.g., lipid droplets) and macromolecules (e.g., proteins and lipids) in lysosomes for maintenance of cell homeostasis
β-Oxidation: The main metabolic process by which fatty acids are broken down in the cell
Chemokine: Any of a group of small signaling proteins that are released by a variety of cells to stimulate the movement of *leukocytes* and attract them to the site of an immune response
Cytokine: Any of a group of small, hormone-like proteins secreted by various cell types that regulate the intensity and duration of immune responses and mediate cell-to-cell communication
Depolarization: Reduction in the difference in electrical charge across a membrane (e.g., between the inside and outside of a cell or a cell compartment, such as a mitochondrion), which can affect numerous cellular functions
Encephalopathy: Any disorder of the brain; syndrome of overall brain dysfunction that can have many different organic and inorganic causes; for example, advanced liver cirrhosis can cause hepatic encephalopathy
Endoplasmic Reticulum (ER): An organelle found in eukaryotic cells that forms an interconnected network of membrane-enclosed sacs or tube-like structures and is connected with the outer membrane of the cell nucleus; the ER serves many functions, including the folding and transport of newly produced proteins that are then delivered to the *Golgi apparatus*
Epigenetic: Pertaining to the regulation of gene expression without altering the DNA sequences; can include chemical modifiations of the DNA or of the proteins (i.e., histones) around which the DNA is wound
Golgi Apparatus: Membrane-enclosed organelle with tube-like structures that plays a role in the transport of newly produced proteins to their destination within the cell or out of the cell; the Golgi apparatus receives proteins packaged into small membrane-enclosed vesicles from the *endoplasmic reticulum* and transports them to their final destinations
Hepatic Stellate Cell (HSC): Cell type found in the liver with several long protrusions that wrap around the *sinusoids.* HSCs play an important role in liver fibrosis; in normal liver, the HSCs are in a resting state but become activated upon liver damage, resulting in cell proliferation and secretion of collagen scar tissue
Hepatorenal Syndrome: The occurrence of kidney failure in patients with liver disease
Kupffer Cell (KC): Specialized immune cells (i.e., macrophages) that reside in the liver and are part of the immune system, particularly inflammatory responses; they play a central role in early stages of alcoholic liver disease
Leukocytes: White blood cells that make up the immune system; they are found throughout the body and include five main types, one of which are the monocytes/*macrophages*
Lipophagy: The selective *autophagy* of lipid droplets
Macrophage: A type of *leukocyte* that act as phagocytes— that is, they ingest and destroy bacteria, foreign particles, and dead or diseased cells or other degenerating material in the body; they also release signaling molecules involved in the immune response
Parenchymal Cells: The distinguishing or specific cells of an organ or gland that are contained in and supported by the connective tissue; parenchymal cells of the liver are the hepatocytes
Peroxisome: A membrane-enclosed organelle found in many eukaryotic cells that contains various enzymes needed for the formation and degradation of hydrogen peroxide (H_2_O_2_); plays a role in breaking down fatty acids and detoxifying various molecules
Portal Hypertension: Elevated blood pressure in the blood system supplying the liver (i.e., portal system); occurs in cirrhosis and other conditions that cause blockage of the portal vein
Promoter: A region of DNA located in front of a gene that regulates and marks the starting point for gene transcription
Proteolytic: Pertaining to or causing the breakdown of proteins (i.e., proteolysis)
Reactive Oxygen Species (ROS): Highly reactive chemical molecules containing oxygen, such as hydrogen peroxide or superoxide, that are formed as natural byproducts of various metabolic reactions but whose levels can increase during times of environmental stress; excess levels of ROS can damage macromolecules (e.g., proteins or DNA)
Sinusoid: A small, thin-walled blood vessel characterized by open pores between the cells lining the vessel, allowing small and medium-sized proteins to readily enter and leave the bloodstream; in the liver, *Kupffer cells* are located inside the sinusoids
Space of Disse: In the liver, the small space that separates the walls of the *sinusoids* from the *parenchymal cells* (i.e., the hepatocytes)
Ubiquitin-Proteasome System: A system comprising multiple components that identifies and degrades unwanted proteins in all cells; is involved in cell growth and differentiation, cell death (i.e., apoptosis), and stress and immune responses
Vacuole: A clear space within a cell that may surround an engulfed foreign particle and may degrade or digest that particle

## References

[b1-arcr-38-2-147] Addolorato G, Capristo E, Greco AV (1997). Energy expenditure, substrate oxidation, and body composition in subjects with chronic alcoholism: New findings from metabolic assessment. Alcoholism: Clinical and Experimental Research.

[b2-arcr-38-2-147] Addolorato G, Capristo E, Greco AV (1998). Influence of chronic alcohol abuse on body weight and energy metabolism: Is excess ethanol consumption a risk factor for obesity or malnutrition?. Journal of Internal Medicine.

[b3-arcr-38-2-147] Addolorato G, Mirijello A, Barrio P, Gual A (2016). Treatment of alcohol use disorders in patients with alcoholic liver disease. Journal of Hepatology.

[b4-arcr-38-2-147] Ambade A, Mandrekar P (2012). Oxidative stress and inflammation: Essential partners in alcoholic liver disease. International Journal of Hepatology.

[b5-arcr-38-2-147] Anstee QM, Seth D, Day CP (2016). Genetic factors that affect risk of alcoholic and nonalcoholic fatty liver disease. Gastroenterology.

[b6-arcr-38-2-147] Aragon CM, Rogan F, Amit Z (1992). Ethanol metabolism in rat brain homogenates by a catalase-H_2_O_2_ system. Biochemical Pharmacology.

[b7-arcr-38-2-147] Bala S, Petrasek J, Mundkur S (2012). Circulating microRNAs in exosomes indicate hepatocyte injury and inflammation in alcoholic, drug-induced, and inflammatory liver diseases. Hepatology.

[b8-arcr-38-2-147] Becker U, Deis A, Sorensen TI (1996). Prediction of risk of liver disease by alcohol intake, sex, and age: A prospective population study. Hepatology.

[b9-arcr-38-2-147] Bergheim I, McClain CJ, Arteel GE (2005). Treatment of alcoholic liver disease. Digestive Diseases.

[b10-arcr-38-2-147] Bode C, Bode JC (2003). Effect of alcohol consumption on the gut. Best Practices & Research. Clinical Gastroenterology.

[b11-arcr-38-2-147] Brooks PJ, Zakhari S (2014). Acetaldehyde and the genome: Beyond nuclear DNA adducts and carcinogenesis. Environmental and Molecular Mutagenesis.

[b12-arcr-38-2-147] Burza MA, Molinaro A, Attilia ML (2014). PNPLA3 I148M (rs738409) genetic variant and age at onset of at-risk alcohol consumption are independent risk factors for alcoholic cirrhosis. Liver International.

[b13-arcr-38-2-147] Chen LH, Xi S, Cohen DA (1995). Liver antioxidant defenses in mice fed ethanol and the AIN-76A diet. Alcohol.

[b14-arcr-38-2-147] Dilger K, Metzler J, Bode JC, Klotz U (1997). CYP2E1 activity in patients with alcoholic liver disease. Journal of Hepatology.

[b15-arcr-38-2-147] Donnadieu-Rigole H, Olive L, Nalpas B (2017). Follow-up of alcohol consumption after liver transplantation: Interest of an addiction team?. Alcoholism: Clinical and Experimental Research.

[b16-arcr-38-2-147] Dong X, Liu H, Chen F (2014). MiR-214 promotes the alcohol-induced oxidative stress via down-regulation of glutathione reductase and cytochrome P450 in liver cells. Alcoholism: Clinical and Experimental Research.

[b17-arcr-38-2-147] Donohue TM (2007). Alcohol-induced steatosis in liver cells. World Journal of Gastroenterology.

[b18-arcr-38-2-147] Donohue TM, Osna NA, Trambly CS (2012). Early growth response-1 contributes to steatosis development after acute ethanol administration. Alcoholism: Clinical and Experimental Research.

[b19-arcr-38-2-147] Donohue TM, Tuma DJ, Sorrell MF (1983). Acetaldehyde adducts with proteins: Binding of [14C]acetaldehyde to serum albumin. Archives of Biochemistry and Biophysics.

[b20-arcr-38-2-147] European Association for the Study of the Liver (2012). EASL clinical practical guidelines: Management of alcoholic liver disease. Journal of Hepatology.

[b21-arcr-38-2-147] Fang YZ, Yang S, Wu G (2002). Free radicals, antioxidants, and nutrition. Nutrition.

[b22-arcr-38-2-147] Frazier TH, Stocker AM, Kershner NA (2011). Treatment of alcoholic liver disease. Therapeutic Advances in Gastroenterology.

[b23-arcr-38-2-147] Frezza M, di Padova C, Pozzato G (1990). High blood alcohol levels in women: The role of decreased gastric alcohol dehydrogenase activity and first-pass metabolism. New England Journal of Medicine.

[b24-arcr-38-2-147] Friedman SL (2000). Molecular regulation of hepatic fibrosis, an integrated cellular response to tissue injury. Journal of Biological Chemistry.

[b25-arcr-38-2-147] Friedman SL (2008). Hepatic stellate cells: Protean, multifunctional, and enigmatic cells of the liver. Physiological Reviews.

[b26-arcr-38-2-147] Fujita T, Soontrapa K, Ito Y (2016). Hepatic stellate cells relay inflammation signaling from sinusoids to parenchyma in mouse models of immune-mediated hepatitis. Hepatology.

[b27-arcr-38-2-147] Gale M, Foy EM (2005). Evasion of intracellular host defence by hepatitis C virus. Nature.

[b28-arcr-38-2-147] Galli A, Pinaire J, Fischer M (2001). The transcriptional and DNA binding activity of peroxisome proliferator-activated receptor alpha is inhibited by ethanol metabolism: A novel mechanism for the development of ethanol-induced fatty liver. Journal of Biological Chemistry.

[b29-arcr-38-2-147] Ganesan M, Natarajan SK, Zhang J (2016). Role of apoptotic hepatocytes in HCV dissemination: Regulation by acetaldehyde. American Journal of Physiology: Gastrointestinal and Liver Physiology.

[b30-arcr-38-2-147] Ganesan M, Zhang J, Bronich T (2015). Acetaldehyde accelerates HCV-induced impairment of innate immunity by suppressing methylation reactions in liver cells. American Journal of Physiology: Gastrointestinal and Liver Physiology.

[b31-arcr-38-2-147] Gitto S, Vitale G, Villa E, Andreone P (2014). Update on alcohol and viral hepatitis. Journal of Clinical and Translational Hepatology.

[b32-arcr-38-2-147] Halsted CH (2004). Nutrition and alcoholic liver disease. Seminars in Liver Disease.

[b33-arcr-38-2-147] Ishak KG, Zimmerman HJ, Ray MB (1991). Alcoholic liver disease: Pathologic, pathogenetic, and clinical aspects. Alcoholism: Clinical and Experimental Research.

[b34-arcr-38-2-147] Jones AL, Zakim D, Boyer TD (1996). Anatomy of the normal liver. Hepatology: A Textbook of Liver Disease, Third Edition.

[b35-arcr-38-2-147] Kang L, Chen X, Sebastian BM (2007). Chronic ethanol and triglyceride turnover in white adipose tissue in rats: Inhibition of the anti-lipolytic action of insulin after chronic ethanol contributes to increased triglyceride degradation. Journal of Biological Chemistry.

[b36-arcr-38-2-147] Keating GM (2015). Ledipasvir/sofosbuvir: A review of its use in chronic hepatitis C. Drugs.

[b37-arcr-38-2-147] Kenney WC (1982). Acetaldehyde adducts of phospholipids. Alcoholism: Clinical and Experimental Research.

[b38-arcr-38-2-147] Khan KN, Yatsuhashi H (2000). Effect of alcohol consumption on the progression of hepatitis C virus infection and risk of hepatocellular carcinoma in Japanese patients. Alcohol and Alcoholism.

[b39-arcr-38-2-147] Kharbanda KK, Mailliard ME, Baldwin CR (2007). Betaine attenuates alcoholic steatosis by restoring phosphatidylcholine generation via the phosphatidylethanolamine methyltransferase pathway. Journal of Hepatology.

[b40-arcr-38-2-147] Kharbanda KK, McVicker DL, Zetterman RK, Donohue TM (1995). Ethanol consumption reduces the proteolytic capacity and protease activities of hepatic lysosomes. Biochimica et Biophysica Acta.

[b41-arcr-38-2-147] Kharbanda KK, McVicker DL, Zetterman RK, Donohue TM (1996). Ethanol consumption alters trafficking of lysosomal enzymes and affects the processing of procathepsin L in rat liver. Biochimica et Biophysica Acta.

[b42-arcr-38-2-147] Kharbanda KK, Todero SL, Ward BW (2009). Betaine administration corrects ethanol-induced defective VLDL secretion. Molecular and Cellular Biochemistry.

[b43-arcr-38-2-147] Kim MS, Ong M, Qu X (2016). Optimal management for alcoholic liver disease: Conventional medications, natural therapy or combination?. World Journal of Gastroenterology.

[b44-arcr-38-2-147] Kirpich IA, Miller ME, Cave MC (2016). Alcoholic liver disease: Update on the role of dietary fat. Biomolecules.

[b45-arcr-38-2-147] Klatsky AL, Armstrong MA (1992). Alcohol, smoking, coffee, and cirrhosis. American Journal of Epidemiology.

[b46-arcr-38-2-147] Lefkowitch JH (2005). Morphology of alcoholic liver disease. Clinics in Liver Disease.

[b47-arcr-38-2-147] Levy RE, Catana AM, Durbin-Johnson B (2015). Ethnic differences in presentation and severity of alcoholic liver disease. Alcoholism: Clinical and Experimental Research.

[b48-arcr-38-2-147] Lieber CS (2000). Alcoholic liver disease: New insights in pathogenesis lead to new treatments. Journal of Hepatology.

[b49-arcr-38-2-147] Lieber CS (2004). Alcoholic fatty liver: Its pathogenesis and mechanism of progression to inflammation and fibrosis. Alcohol.

[b50-arcr-38-2-147] Lieber CS, DeCarli LM (1968). Ethanol oxidation by hepatic microsomes: Adaptive increase after ethanol feeding. Science.

[b51-arcr-38-2-147] Lucey MR (2014). Liver transplantation for alcoholic liver disease. Nature Reviews. Gastroenterology & Hepatology.

[b52-arcr-38-2-147] Masson S, Emmerson I, Henderson E (2014). Clinical but not histological factors predict long-term prognosis in patients with histologically advanced non-decompensated alcoholic liver disease. Liver International.

[b53-arcr-38-2-147] Mathurin P, Deng QG, Keshavarzian A (2000). Exacerbation of alcoholic liver injury by enteral endotoxin in rats. Hepatology.

[b54-arcr-38-2-147] Mathurin P, Duchatelle V, Ramond MJ (1996). Survival and prognostic factors in patients with severe alcoholic hepatitis treated with prednisolone. Gastroenterology.

[b55-arcr-38-2-147] Mathurin P, Louvet A, Duhamel A (2013). Prednisolone with vs without pentoxifylline and survival of patients with severe alcoholic hepatitis: A randomized clinical trial. JAMA.

[b56-arcr-38-2-147] Mauch TJ, Donohue TM, Zetterman RK (1986). Covalent binding of acetaldehyde selectively inhibits the catalytic activity of lysine-dependent enzymes. Hepatology.

[b57-arcr-38-2-147] McCartney EM, Semendric L, Helbig KJ (2008). Alcohol metabolism increases the replication of hepatitis C virus and attenuates the antiviral action of interferon. Journal of Infectious Diseases.

[b58-arcr-38-2-147] Meier P, Seitz HK (2008). Age, alcohol metabolism and liver disease. Current Opinion in Clinical Nutrition and Metabolic Care.

[b59-arcr-38-2-147] Mendenhall C, Roselle GA, Gartside P, Moritz T (1995). Relationship of protein calorie malnutrition to alcoholic liver disease: A reexamination of data from two Veterans Administration Cooperative Studies. Alcoholism: Clinical and Experimental Research.

[b60-arcr-38-2-147] Mohammad MK, Zhou Z, Cave M (2012). Zinc and liver disease. Nutrition in Clinical Practice.

[b61-arcr-38-2-147] Mumenthaler MS, Taylor JL, O’Hara R, Yesavage JA (1999). Gender differences in moderate drinking effects. Alcohol Research & Health.

[b62-arcr-38-2-147] Nagy LE, Ding WX, Cresci G (2016). Linking pathogenic mechanisms of alcoholic liver disease with clinical phenotypes. Gastroenterology.

[b63-arcr-38-2-147] Osna NA, White RL, Krutik VM (2008). Proteasome activation by hepatitis C core protein is reversed by ethanol-induced oxidative stress. Gastroenterology.

[b64-arcr-38-2-147] Otani K, Korenaga M, Beard MR (2005). Hepatitis C virus core protein, cytochrome P450 2E1, and alcohol produce combined mitochondrial injury and cytotoxicity in hepatoma cells. Gastroenterology.

[b65-arcr-38-2-147] Pavlov CS, Casazza G, Semenistaia M (2016). Ultrasonography for diagnosis of alcoholic cirrhosis in people with alcoholic liver disease. Cochrane Database of Systematic Reviews.

[b66-arcr-38-2-147] Ramond MJ, Poynard T, Rueff B (1992). A randomized trial of prednisolone in patients with severe alcoholic hepatitis. New England Journal of Medicine.

[b67-arcr-38-2-147] Roberts BJ, Song BJ, Soh Y (1995). Ethanol induces CYP2E1 by protein stabilization. Role of ubiquitin conjugation in the rapid degradation of CYP2E1. Journal of Biological Chemistry.

[b68-arcr-38-2-147] Ruhl CE, Everhart JE (2005). Joint effects of body weight and alcohol on elevated serum alanine aminotransferase in the United States population. Clinical Gastroenterology and Hepatology.

[b69-arcr-38-2-147] Schiodt FV, Lee WM, Bondesen S (2002). Influence of acute and chronic alcohol intake on the clinical course and outcome in acetaminophen overdose. Alimentary Pharmacology & Therapeutics.

[b70-arcr-38-2-147] Seronello S, Montanez J, Presleigh K (2011). Ethanol and reactive species increase basal sequence heterogeneity of hepatitis C virus and produce variants with reduced susceptibility to antivirals. PLoS One.

[b71-arcr-38-2-147] Seronello S, Sheikh MY, Choi J (2007). Redox regulation of hepatitis C in nonalcoholic and alcoholic liver. Free Radical Biology & Medicine.

[b72-arcr-38-2-147] Shearn CT, Fritz KS, Shearn AH (2016). Deletion of GSTA4-4 results in increased mitochondrial post-translational modification of proteins by reactive aldehydes following chronic ethanol consumption in mice. Redox Biology.

[b73-arcr-38-2-147] Singal AK, Bashar H, Anand BS (2012). Outcomes after liver transplantation for alcoholic hepatitis are similar to alcoholic cirrhosis: Exploratory analysis from the UNOS database. Hepatology.

[b74-arcr-38-2-147] Singal AK, Chaha KS, Rasheed K, Anand BS (2013). Liver transplantation in alcoholic liver disease current status and controversies. World Journal of Gastroenterology.

[b75-arcr-38-2-147] Siu L, Foont J, Wands JR (2009). Hepatitis C virus and alcohol. Seminars in Liver Disease.

[b76-arcr-38-2-147] Sofair AN, Barry V, Manos MM (2010). The epidemiology and clinical characteristics of patients with newly diagnosed alcohol-related liver disease: Results from population-based surveillance. Journal of Clinical Gastroenterology.

[b77-arcr-38-2-147] Stickel F, Hampe J (2012). Genetic determinants of alcoholic liver disease. Gut.

[b78-arcr-38-2-147] Stickel F, Hoehn B, Schuppan D, Seitz HK (2003). Review article: Nutritional therapy in alcoholic liver disease. Alimentary Pharmacology & Therapeutics.

[b79-arcr-38-2-147] Strader DB, Bacon BR, Lindsay KL (8262). Use of complementary and alternative medicine in patients with liver disease. American Journal of Gastroenterology.

[b80-arcr-38-2-147] Sugimoto T, Yamashita S, Ishigami M (2002). Decreased microsomal triglyceride transfer protein activity contributes to initiation of alcoholic liver steatosis in rats. Journal of Hepatology.

[b81-arcr-38-2-147] Suraweera DB, Weeratunga AN, Hu RW (2015). Alcoholic hepatitis: The pivotal role of Kupffer cells. World Journal of Gastrointestinal Pathophysiology.

[b82-arcr-38-2-147] Szabo G, Aloman C, Polyak SJ (2006). Hepatitis C infection and alcohol use: A dangerous mix for the liver and antiviral immunity. Alcoholism: Clinical and Experimental Research.

[b83-arcr-38-2-147] Teli MR, Day CP, Burt AD (1995). Determinants of progression to cirrhosis or fibrosis in pure alcoholic fatty liver. Lancet.

[b84-arcr-38-2-147] Thomes PG, Osna NA, Davis JS, Donohue TM (2013). Cellular steatosis in ethanol oxidizing-HepG2 cells is partially controlled by the transcription factor, early growth response-1. International Journal of Biochemistry & Cell Biology.

[b85-arcr-38-2-147] Thursz M, Forrest E, Roderick P (2015). The clinical effectiveness and cost-effectiveness of STeroids Or Pentoxifylline for Alcoholic Hepatitis (STOPAH): A 2 × 2 factorial randomised controlled trial. Health Technology Assessment.

[b86-arcr-38-2-147] Tsukamoto H, Machida K, Dynnyk A, Mkrtchyan H (2009). “Second hit” models of alcoholic liver disease. Seminars in Liver Disease.

[b87-arcr-38-2-147] Tuma DJ, Thiele GM, Xu D (1996). Acetaldehyde and malondialdehyde react together to generate distinct protein adducts in the liver during long-term ethanol administration. Hepatology.

[b88-arcr-38-2-147] Venkatesan S, Ward RJ, Peters TJ (1988). Effect of chronic ethanol feeding on the hepatic secretion of very-low-density lipoproteins. Biochimica et Biophysica Acta.

[b89-arcr-38-2-147] Wang ZG, Dou XB, Zhou ZX, Song ZY (2016). Adipose tissue-liver axis in alcoholic liver disease. World Journal of Gastrointestinal Pathophysiology.

[b90-arcr-38-2-147] Wei X, Shi X, Zhong W (2013). Chronic alcohol exposure disturbs lipid homeostasis at the adipose tissue-liver axis in mice: Analysis of triacylglycerols using high-resolution mass spectrometry in combination with in vivo metabolite deuterium labeling. PLoS One.

[b91-arcr-38-2-147] World Health Organization (WHO) (2014). Global Status Report on Alcohol and Health.

[b92-arcr-38-2-147] Xu A, Wang Y, Keshaw H (2003). The fat-derived hormone adiponectin alleviates alcoholic and nonalcoholic fatty liver diseases in mice. Journal of Clinical Investigation.

[b93-arcr-38-2-147] You M, Crabb DW (2004). Recent advances in alcoholic liver disease. II. Minireview: Molecular mechanisms of alcoholic fatty liver. American Journal of Physiology: Gastrointestinal and Liver Physiology.

[b94-arcr-38-2-147] Zakhari S (2013). Bermuda Triangle for the liver: Alcohol, obesity, and viral hepatitis. Journal of Gastroenterology and Hepatology.

[b95-arcr-38-2-147] Zakhari S, Li TK (2007). Determinants of alcohol use and abuse: Impact of quantity and frequency patterns on liver disease. Hepatology.

[b96-arcr-38-2-147] Zhao M, Matter K, Laissue JA, Zimmermann A (1996). Copper/zinc and manganese superoxide dismutases in alcoholic liver disease: Immunohistochemical quantitation. Histology and Histopathology.

[b97-arcr-38-2-147] Zhong Z, Ramshesh VK, Rehman H (2014). Acute ethanol causes hepatic mitochondrial depolarization in mice: Role of ethanol metabolism. PLoS One.

